# Integrative analysis of immune and microbial subtypes predicts immunotherapy response in stomach adenocarcinoma

**DOI:** 10.1128/spectrum.02151-25

**Published:** 2026-02-09

**Authors:** Yumeng Zhang, Huakai Wen, Xianfang Tang, Yuhua Yao

**Affiliations:** 1 School of Mathematics and Statistics, Hainan Normal University 12389, Haikou, China; 2 School of Computer Science and Artificial Intelligence, Wuhan Textile University 216798, Wuhan, China; 3 Key Laboratory of Data Science and Intelligence Education, Ministry of Education, Hainan Normal University 12389, Haikou, China; 4 Key Laboratory of Computational Science and Application of Hainan Province, Hainan Normal University 12389, Haikou, China; Cleveland Clinic Lerner Research Institute, Cleveland, Ohio, USA

**Keywords:** stomach adenocarcinoma, tumor immune microenvironment, intratumoral microbiota, immune checkpoint blockade, machine learning

## Abstract

The tumor immune microenvironment and intratumoral microbiota play critical roles in cancer progression and immunotherapy response, yet their integrated functions in stomach adenocarcinoma (STAD) are not well understood. This study conducted a multi-omics analysis of transcriptomic and microbiome data from 348 patients with STAD. Using the ImmuCellAI algorithm, immune cell infiltration (ICI) was estimated, and non-negative matrix factorization classified samples into three immune subtypes (INC-1, INC-2, and INC-3). Differential expression analysis identified immune-related signature genes enriched in immune signaling pathways. Tumor mutational burden, microsatellite instability, immune checkpoint gene expression, and drug sensitivity were compared across subtypes. Microbiome clustering identified three subtypes (MC-1, MC-2, and MC-3), with associations to immune infiltration and microbial composition. The immune subtypes showed distinct patterns of ICI, clinical stage, and gene expression, with differentially expressed genes enriched in immune and tumor-related pathways. Microbiome subtypes exhibited unique diversity metrics and associations with the immune microenvironment. Integration of immune and microbial data improved immune checkpoint blockade (ICB) prediction, with genera like *Staphylococcus* and *Ralstonia* correlating with immune genes such as CD22, VIPR2, and FLT3. These findings provide insights into ICB response and support more precise immunotherapy strategies for STAD.

**IMPORTANCE:**

Deciphering the interactions between the tumor immune microenvironment and the intratumoral microbiota is crucial for advancing precision immunotherapy in stomach adenocarcinoma (STAD). In this study, we present an integrative multi-omics framework that stratifies patients into distinct immune and microbial subtypes, uncovering their associations with immunogenomic profiles, immune cell infiltration patterns, and clinical features. Notably, we identify specific microbial genera correlated with immune-related gene expression and immune checkpoint blockade responsiveness. These findings provide novel insights into the immune–microbiome axis in STAD and underscore the potential of integrative multi-omics approaches to enhance patient stratification and guide more effective immunotherapeutic strategies.

## INTRODUCTION

Stomach adenocarcinoma (STAD), the main subtype of gastric cancer, remains a global health challenge despite a declining incidence (1–3). Advanced STAD is often diagnosed late, and prognosis is poor due to resistance to conventional therapies and limited efficacy of current treatments (4). Growing evidence highlights the tumor microenvironment (TME), particularly immune cell infiltration (ICI), as a key factor in tumor progression and therapeutic response (5–9). Consequently, there is an urgent need to identify novel biomarkers and robust stratification frameworks to guide precision medicine strategies.

The TME comprises a complex network of cellular and non-cellular components, including immune cells, fibroblasts, endothelial cells, and the extracellular matrix, all of which collectively influence tumor growth, metastasis, and treatment outcomes  (10–13).  Among these, tumor-infiltrating immune cells (TIICs) have emerged as key modulators of cancer immunity, exhibiting both pro- and anti-tumorigenic effects (14, 15). The presence, abundance, and spatial distribution of various immune cell subsets—such as cytotoxic T lymphocytes, regulatory T cells, macrophages, and dendritic cells—profoundly impact tumor immunogenicity and response to immunotherapeutic interventions, particularly immune checkpoint blockade (ICB) (16–20).

Recent advances in high-throughput sequencing technologies and computational deconvolution algorithms have facilitated the identification of immune subtypes across various cancer types (21–23). However, studies specifically characterizing immune infiltration patterns in STAD, along with their clinical and molecular implications, remain limited. In parallel, the tumor-associated microbiome has emerged as a key modulator of the immune microenvironment, influencing tumor progression, immune evasion, and response to ICB therapies (24–26). Despite these insights, the complex interplay between microbial composition and the immune landscape in STAD is not yet fully understood.

Mounting evidence also highlights the tumor-resident microbiome as a critical determinant of host immune regulation  (27–30). Microorganisms present within tumor tissues and the surrounding microenvironment can influence antigen presentation, cytokine signaling, and immune cell recruitment, thereby shaping the immunological tone of the TME (31, 32). In gastrointestinal malignancies such as STAD, microbial communities may engage in direct interactions with epithelial and immune cells, impacting tumorigenesis and therapeutic efficacy (33). Nevertheless, the mechanistic relationship between the tumor microbiome and immune infiltration in STAD remains poorly characterized.

In this study, we performed an integrative multi-omics analysis of STAD using transcriptomic and microbial sequencing data from The Cancer Genome Atlas (TCGA). Our objectives were to (i) characterize the immune infiltration landscape and identify robust immune subtypes using non-negative matrix factorization (NMF); (ii) investigate subtype-specific differences in immune-related gene expression, tumor mutational burden (TMB), microsatellite instability (MSI), immune checkpoint expression, and drug sensitivity; (iii) define microbial subtypes based on intratumoral microbiome composition and examine their immunological associations; and (iv) construct predictive models integrating immune and microbial features to assess their predictive value for ICB response. Collectively, our findings offer a comprehensive view of the immune–microbial–tumor axis in STAD and propose novel biomarkers and therapeutic targets to advance personalized immunotherapy.

## MATERIALS AND METHODS

### Data acquisition and preprocessing

Transcriptomic, clinical, and survival data for patients with STAD (*n* = 348) were retrieved from the TCGA-STAD database. Matched tumor-associated microbiome abundance profiles were obtained from the pan-cancer resource published by Haziza et al. (34). This data set was processed through a two-step decontamination pipeline comprising: (i) computational decontamination using the decontam R package (v1.14.0) in “frequency” mode with DNA/RNA concentrations and batch information (P-threshold = 0.1) and (ii) manual curation against known human commensals and pathogens from established databases and literature. The final decontaminated tables from Haziza et al. were used for all downstream analyses.

### ICI and subtype clustering

ICI was estimated using the ImmuCellAI algorithm (35). Unsupervised NMF clustering was performed to define ICI clusters (INCs). The clustering was conducted using the singleCollectionTool (http://www.sxdyc.com/toolGather), with k-values ranging from 2 to 10 evaluated. The optimal k = 3 was selected based on the stability analysis, considering the cophenetic coefficient and the “elbow” in the residual sum of squares plot (Fig. S1). Differences in immune infiltration, clinical features, and survival outcomes were then analyzed across the INC subtypes.

### Immune-related differential gene expression analysis

Differential gene expression was analyzed using the DESeq2 R package on mRNA count data. The design formula used was design = ~ group, and comparisons were made between groups (e.g., INC-1 vs INC-2, INC-1 vs INC-3, INC-2 vs INC-3). Genes with log2(FC) > 0 were considered upregulated in the first group, and those with log2(FC) < 0 in the second. No additional covariates (e.g., stage, sex, or purity) were included. Gene Ontology (GO) and Kyoto Encyclopedia of Genes and Genomes (KEGG) analyses using the DAVID (https://davidbioinformatics.nih.gov/) tool (36). The input gene list consisted of differentially expressed immune-related genes (DEIRGs) from DESeq2, using OFFICIAL_GENE_SYMBOL. The background gene set was all protein-coding genes in Homo sapiens. GO enrichment used GO Direct categories, excluding parent terms, and KEGG pathways were analyzed using the KEGG database. Fisher’s exact test was used for statistical significance, and *P*-values were adjusted for multiple testing using the Benjamini-Hochberg (FDR) method (FDR ≤ 0.05). Functional annotation clustering was applied to reduce redundancy using Kappa statistics in DAVID. The ImmPort gene list (downloaded on 27 February 2025) was used to identify immune-related genes for differential expression analysis. Duplicate entries in the gene symbol column were removed, and the list is provided as Table S1.

### Immune checkpoint, TME scores, and genomic profiling

The expression of key immune checkpoint genes (PDCD1, CTLA4, CD274, LAG3, and TIGIT) was analyzed using FPKM expression data from our data set, comparing their levels across the INC subtypes. These genes were selected for their relevance in immunotherapy studies. The ESTIMATE algorithm (37) was applied to calculate stromal scores and tumor purity. TMB was calculated based on the upper quartile cutoff for mutation counts, and MSI was determined using data from the MSIfromHE GitHub repository (https://github.com/jnkather/MSIfromHE), with histological whole-slide images from TCGA. For genomic profiling, single-nucleotide variant (SNV) profiles were assessed using the maftools R package and visualized through oncoplots to explore immunogenomic heterogeneity.

### Microbial community analysis and integration with immune subtypes

Microbial abundance profiles were preprocessed by removing taxa with >70% zero values, representing taxa present in less than 30% of the samples. The filtered data were normalized to relative abundance. Microbial community subtypes were identified using consensus clustering via the ConsensusClusterPlus package (v1.60.0) in R, applying the Partitioning Around Medoids (PAM) algorithm with Euclidean distance over 500 bootstrap iterations (pItem = 0.8). Clustering was evaluated for k values ranging from 2 to 10. The optimal k = 3 was determined based on the delta area criterion, where the relative change in the area under the cumulative distribution function curve between k and k − 1 was largest (Fig. S2). To ensure the robustness of the results, a sensitivity analysis was also performed by applying Bray-Curtis distance for consensus clustering. The results from clustering using Euclidean distance and Bray-Curtis distance were highly consistent, with an Adjusted Rand Index of 0.724, indicating that the choice of Euclidean distance for clustering did not affect the overall classification of microbial subtypes. Further evaluation of clustering stability was carried out with silhouette scores, which were 0.1103 for Euclidean distance and 0.0894 for Bray-Curtis distance, suggesting that both methods provided reasonable but suboptimal clustering quality. Alpha diversity was calculated using Shannon, Simpson, and Observed Richness indices with the vegan package in R. Beta diversity was visualized using Principal Component Analysis from the FactoMineR package, with 95% confidence ellipses drawn using ggforce. Differences in diversity indices and principal components were assessed using the Kruskal–Wallis test followed by pairwise Wilcoxon rank-sum tests, with *P*-values adjusted using the Benjamini–Hochberg (BH) method. Associations between microbial relative abundance and ICI scores were quantified using Spearman’s rank correlation, with FDR adjustment applied across all taxon-immune cell pairs. A complete table of all associations with *q*-values is provided in Table S2.

### ICB response analysis and machine learning modeling

To explore immune–microbial interactions in ICB response, ICB responder labels were integrated and analyzed across INC and MC subtypes. A Boruta algorithm (38) was applied to identify key features from immune genes and microbial taxa for modeling. To prevent data leakage, all feature selection and standardization procedures were strictly performed within the training folds of a nested fivefold cross-validation scheme, with consistent data splits applied across gene-only, microbe-only, and combined models. Class imbalance in the training set was addressed using the Synthetic Minority Over-sampling Technique. Hyperparameter tuning for six machine learning algorithms (Logistic Regression, Decision Tree, Naïve Bayes, Random Forest, support vector machine [SVM], and XGBoost) was conducted via grid search in the inner cross-validation loop. A fixed random seed (39) ensured reproducibility. Model performance was assessed by the mean AUC from the outer cross-validation folds. In the absence of an independent validation cohort, bootstrap sensitivity analysis (1,000 iterations) was employed to estimate 95% confidence intervals for the AUC and bound model optimism.

### Drug sensitivity profiling

Drug sensitivity data were obtained from the Genomics of Drug Sensitivity in Cancer (GDSC) database (40), covering 198 compounds. Drug response predictions for patient tumors were derived by projecting RNA-seq data (FPKM normalized) onto the GDSC cell-line pharmacogenomics database using the OncoPredict algorithm (available at https://github.com/HuangLabUMN/oncoPredict). The model was trained using GDSC gene expression and drug sensitivity (IC50) data, with gene symbols from tumor samples matched to GDSC gene symbols. The lowest 20% of variable genes were excluded to improve model robustness. No explicit batch correction was applied due to platform differences between the training and test data sets. Predicted drug response (IC50 values) was used as the response metric, with lower values indicating higher drug sensitivity. For comparisons between responders (R) and non-responders (NR) across 198 compounds, univariate screening was performed using Wilcoxon rank-sum tests, with FDR control via the BH procedure. Cliff’s delta was used to quantify effect sizes, with thresholds for interpretation: negligible (|δ| < 0.147), small (0.147 ≤ |δ| < 0.33), medium (0.33 ≤ |δ| ≤ 0.474), and large (|δ| > 0.474). Significant compounds from univariate analysis were further evaluated using multivariate logistic regression, adjusting for potential confounders (INC subtype, tumor purity, tumor stage). Non-metric multidimensional scaling (NMDS) was used to assess overall drug response profiles, calculated with Bray-Curtis distances. Dimensionality reduction was performed using the metaMDS function from the vegan package, with a stress value of 0.18 indicating acceptable dimensionality reduction. Group separation was tested using PERMANOVA with 999 permutations. Comprehensive statistical results, including univariate *P*-values, FDR-adjusted *P*-values, Cliff’s delta effect sizes, and multivariate regression outputs, are available in Tables S4 and S5. Predicted IC50 values for each sample and compound are provided in Table S6.

### Statistical analysis

Statistical analyses and graphical visualizations were performed using R (4.4.0) and Sanger box (https://sangerbox.com/). Comparisons of continuous variables between groups were conducted using the t-test, Kruskal–Wallis, or Wilcoxon rank-sum test. A *P*-value < 0.05 was considered statistically significant (two-tailed).

## RESULTS

### Immune infiltration landscape in the TME of STAD

**Fig 1:**
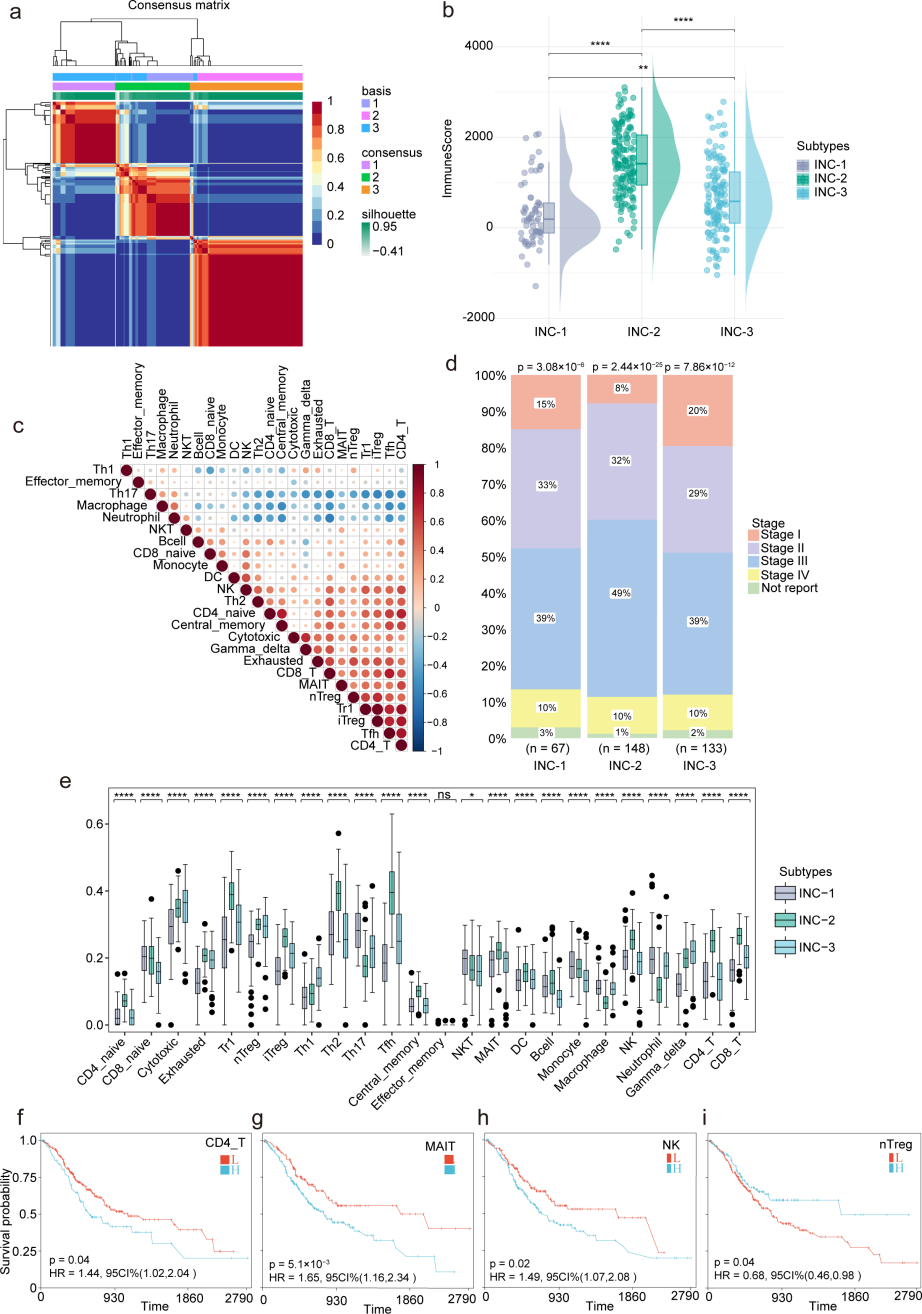
Immune landscape profiling within the TME of STAD. (**a**) Heatmap of ICI clustering using NMF. (**b**) Boxplots showing differences in immune cell scores among INC groups; *P*-values were calculated using the Wilcoxon test. ns indicates no significant difference, * indicates *P* < 0.05, ** indicates *P* < 0.01, *** indicates *P* < 0.001 and **** indicates *P* < 0.0001. (**c**) Correlation heatmap of 24 immune cell types. (**d**) Bar plot of clinical stage distribution across INC groups. (**e**) Boxplots showing differences in infiltration levels of 24 immune cell types among INC groups. (**f–i**) Kaplan–Meier survival curves for CD4_T, MAIT, NK, and nTreg.

The ICI landscape of STAD was analyzed using transcriptomic data from the TCGA-STAD cohort with the ImmuCellAI algorithm. Based on immune infiltration profiles, unsupervised clustering via NMF was performed, classifying samples into three distinct INCs, designated as INC-1, INC-2, and INC-3 (Fig. 1a). Significant differences in ICI scores across the subtypes were observed (Fig. 1b), and a correlation heatmap of 24 immune cell types revealed co-regulation patterns within the TME (Fig. 1c). Clinical staging parameters—T stage (Fig. S3a), N stage (Fig. S3b), and overall pathological stage (Fig. 1d)—were associated with INC subtypes, indicating a potential relationship between patterns of immune infiltration and tumor progression. In-depth analysis of the infiltration levels of 24 immune cell types among the subtypes was conducted, with visualization via boxplots. Statistical comparisons using the Kruskal–Wallis test revealed significant inter-subtype variation in multiple immune cell populations, reinforcing the immunological distinctiveness of each subtype (Fig. 1e). To evaluate the prognostic relevance of TICCs, survival analysis was conducted for various immune subsets (Fig. 1f through i). Notably, high infiltration of natural regulatory T cells (nTregs) was significantly associated with improved overall survival (*P* < 0.05), underscoring the potential prognostic value of specific immune cell populations in STAD.

### Immune regulatory mechanisms and genomic features of distinct immune subtypes in STAD

**Fig 2:**
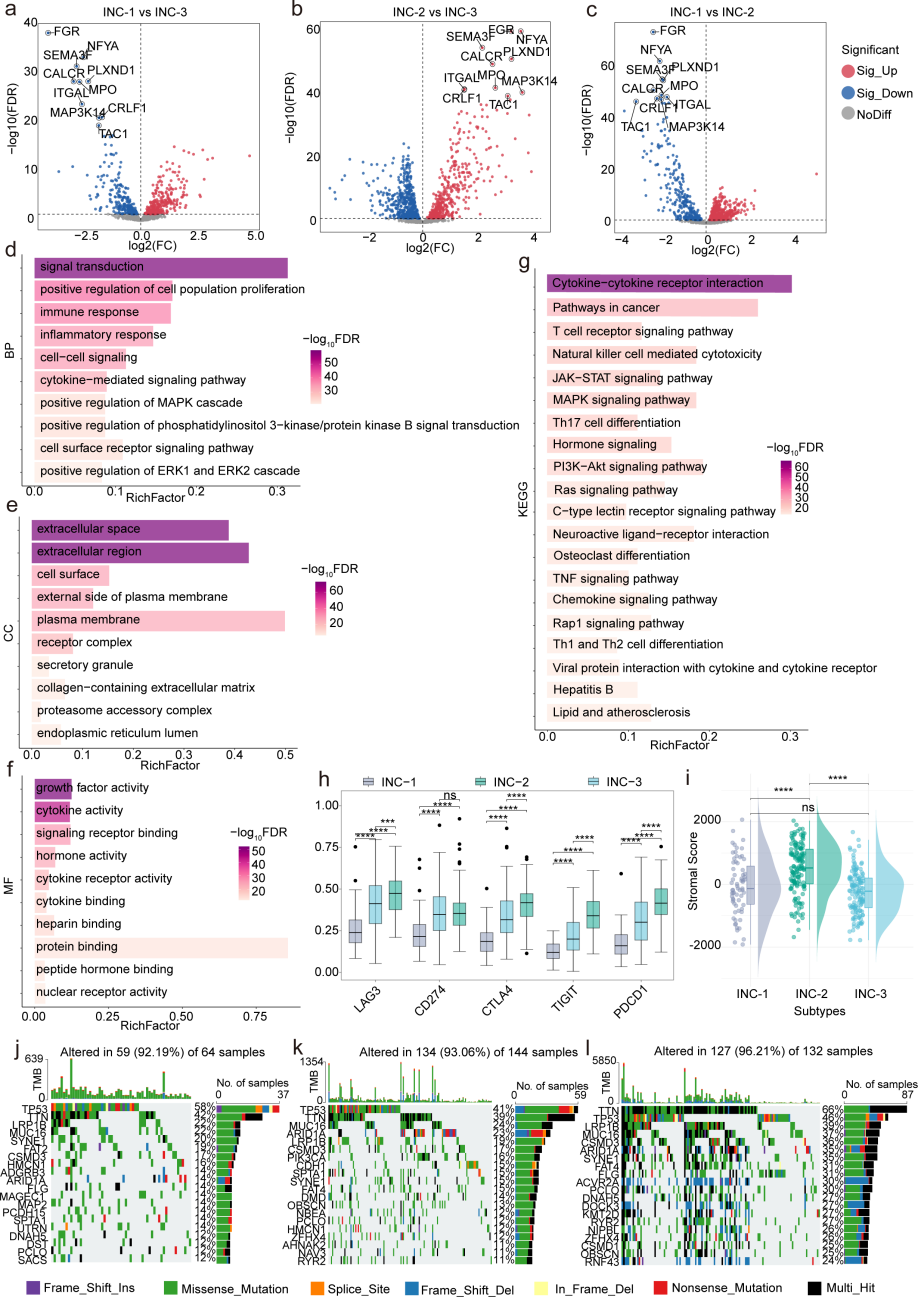
Molecular immune regulation and genomic profiles of different immune subtypes in STAD. (**a–c**) Volcano plots show the pairwise differential expression analyses between the INC-1, INC-2, and INC-3, highlighting significantly upregulated and downregulated genes in each comparison. (**d–g**) Horizontal bar charts display GO and KEGG enrichment. (**h**) Boxplots illustrate the differential expression of immune checkpoint inhibitors among the INC subtypes. (**i**) Boxplots show differences in stromal score among INC subtypes. (**j–l**) Waterfall plots depict SNV profiles across INC subtypes.

To further elucidate the immune regulatory landscape among the identified ICI subtypes, pairwise differential expression analyses were conducted using 1,330 immune-related genes curated from the ImmPort database. Volcano plots (Fig. 2a through c) revealed widespread transcriptional variation in immune gene expression across the subtype comparisons. By integrating the results from all pairwise comparisons, a total of 506 overlapping DEIRGs were identified (Fig. S4a). These DEIRGs may represent core immunological signatures that play pivotal roles in shaping the TME of STAD.

Functional characterization of the DEIRGs was performed via GO and KEGG enrichment analysis using the DAVID tool. Results revealed that DEIRGs are involved in immune regulation, molecular functions, and key pathways. In biological processes (BPs) (Fig. 2d), DEIRGs were enriched in signal transduction (GO:0007165), immune response (GO:0006955), inflammatory response (GO:0006954), cell-cell signaling (GO:0007267), and positive regulation of cell population proliferation (GO:0008284), emphasizing their roles in immune activation and intercellular communication. Moreover, positive regulation of the MAPK cascade, PI3K-Akt signaling, and the ERK1/2 cascade emphasized their involvement in immune and survival pathways. In the cellular component (Fig. 2e), DEIRGs were localized to the extracellular region (GO:0005576), extracellular space (GO:0005615), cell surface (GO:0009986), and external side of the plasma membrane (GO:0009897), indicating roles in extracellular signaling and immune interaction. Regarding molecular functions (Fig. 2f), they were linked to cytokine activity (GO:0005125), growth factor activity (GO:0008083), signaling receptor binding (GO:0005102), and cytokine receptor binding, emphasizing their roles in immune ligand–receptor interactions and signal transduction. KEGG analysis (Fig. 2g) showed that DEIRGs were enriched in inflammation- and immunity-related pathways, including cytokine–cytokine receptor interaction (hsa04060), T-cell receptor (TCR) signaling pathway (hsa04660), natural killer cell-mediated cytotoxicity (hsa04650), JAK-STAT signaling (hsa04630), and TNF signaling (hsa04668). Additionally, DEIRGs were also involved in broader oncogenic and immune pathways such as pathways in cancer (hsa05200), MAPK, PI3K-Akt, Ras signaling, Th1/Th2/Th17 differentiation, and viral protein-cytokine receptor interaction, suggesting their central role in integrating tumor immune microenvironment (TIME) signaling networks.

The expression levels of five ICIs (PDCD1, CTLA4, LAG3, CD274, and TIGIT) were assessed across the three INC subtypes, revealing significant differences (*P* < 0.05) (Fig. 2h), suggesting distinct immune evasion profiles and potential differential responses to ICB therapies. The ESTIMATE algorithm further showed significant differences in stromal scores (Fig. 2i) and tumor purity (Fig. S4b) among the subtypes (*P* < 0.05), indicating heterogeneity in stromal infiltration and tumor cellularity.

The mutational landscape was characterized by using the maftools R package to compute TMB and MSI status, followed by visualization of their distribution across the INC subtypes. As shown in Fig. S4c and d, both TMB and MSI status exhibited significant subtype-specific differences, suggesting varying levels of tumor immunogenicity. These observations are consistent with previous studies reporting that immune-related molecular subtypes in STAD are associated with distinct TMB and MSI profiles, which may shape the TIME and affect responsiveness to immunotherapy (39, 41, 42). Moreover, SNV profiles were analyzed using oncoplots (Fig. 2j through l), revealing distinct mutational patterns and frequently mutated genes across subtypes. Together, these results suggest that immune infiltration patterns and genomic alterations influence the immunogenic landscape and may impact immunotherapy responsiveness in STAD.

### Microbial community profiling reveals distinct clusters and their immunological associations in STAD

**Fig 3:**
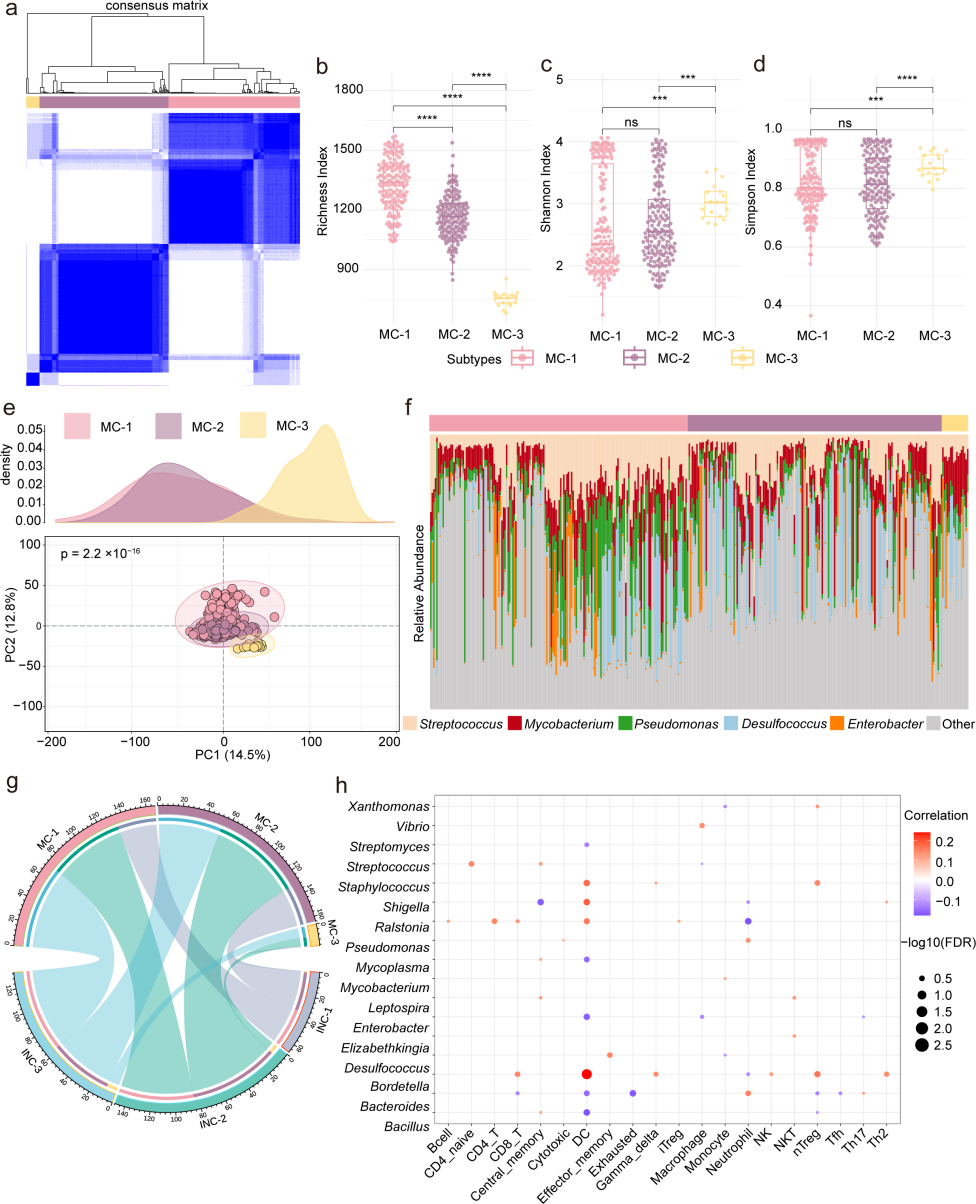
Characterization of tumor-associated microbiota uncovers distinct groups linked to immune features in STAD. (**a**) Consensus clustering identified three microbial subtypes: MC-1, MC-2, and MC-3. (**b–d**) Boxplots show intergroup differences in microbial richness, Shannon, and Simpson diversity index. (**e**) PCoA was performed to visualize the distribution and separation among the MC subtypes. ns indicates no significant difference, * indicates *P* < 0.05, ** indicates *P* < 0.01, *** indicates *P* < 0.001 and **** indicates p < 0.0001. (**f**) A clustered bar plot displays the distribution of key genera—including *Streptococcus*, *Mycobacterium*, *Pseudomonas*, *Desulfococcus*, *Enterobacter*, and others—across MC subtypes. (**g**) A chord diagram visualizes the relationships between INC and MC subtypes, highlighting the distribution and overlap of samples across the two classification systems. (**h**) A bubble plot shows correlations between 17 microbial genera and 24 immune cell types, with bubble size and color indicating correlation strength and direction.

We investigated the interplay between tumor microbiota and the immune microenvironment in STAD by profiling microbes in the same patient cohort used for immune analyses. Unsupervised consensus clustering identified three distinct microbial clusters (MC-1, MC-2, and MC-3) based on the relative abundance of microbial taxa (where MC denotes Microbial Consensus cluster) (Fig. 3a). Alpha-diversity indices, including Shannon and Simpson indices, revealed significant differences in microbial diversity among the clusters, suggesting divergent ecological landscapes (Fig. 3b through d). Furthermore, beta-diversity was assessed using Principal Coordinates Analysis (PCoA), performed with the vegan package in R based on Bray-Curtis dissimilarity (Fig. 3e). The PCoA plots demonstrated clear separation among the clusters with statistical significance (*P* < 0.05), further supporting the robustness of the microbial stratification. Stacked bar charts displayed the top five most abundant microbial genera, highlighting compositional shifts among the clusters (Fig. 3f). A chord diagram (Fig. 3g) illustrated the distributional relationships between the MC and INC clusters, revealing potential interactions. Correlation analysis was performed between the top 20 microbial genera and 24 immune cell types, and results were visualized as a bubble plot (Fig. 3h). This analysis revealed multiple significant associations, suggesting that specific microbial taxa may influence ICI patterns and immune modulation within the TME.

### Integration of immune and microbial signatures reveals predictors of ICB response in STAD

**Fig 4:**
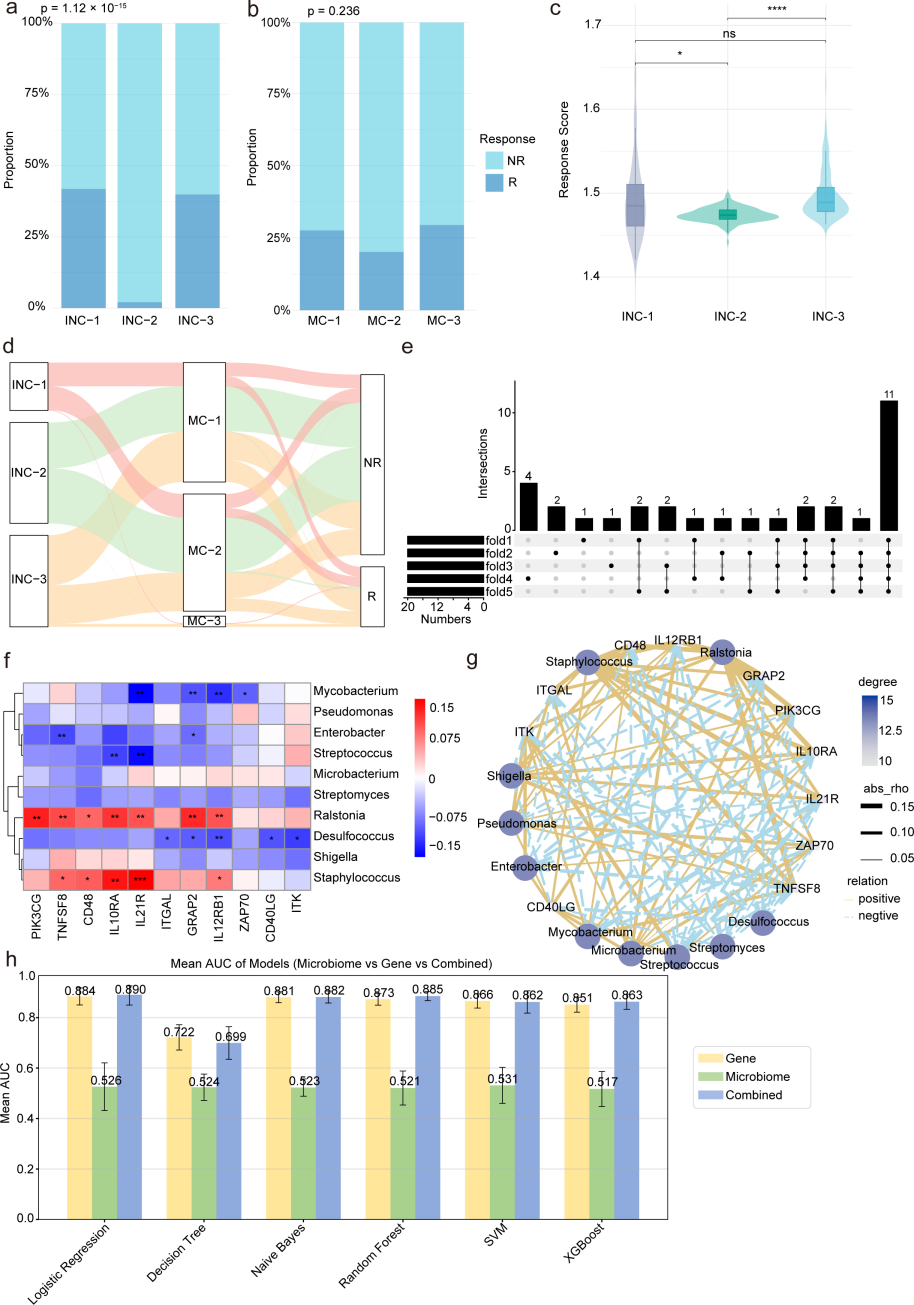
A comprehensive integration of immune cell profiles and microbial signatures uncovers potential predictors of ICB responsiveness in STAD. (**a and b**) Distribution of ICB response across INC and MC subtypes in STAD. (**c**) Boxplot illustrates the differences in ICB response scores among INC subtypes. ns indicates no significant difference, * indicates *P* < 0.05, ** indicates *P* < 0.01, *** indicates *P* < 0.001 and **** indicates *P* < 0.0001. (**d**) Sankey diagram depicting the distributional relationships among INC subtypes, MC subtypes, and ICB response status R vs. NR. (**e**) UpSet plot showing the overlap of gene features selected across cross-validation folds, highlighting the consistency and variation in immune-related gene selection. (**f and g**) Heatmap and network diagram illustrating the relationships between the top 10 microbial genera (based on relative abundance) and the top 11 immune-related genes selected by intersecting features across cross-validation folds. (**h**) Bar plots depicting the AUC values of gene-only, microbe-only, and combined models across six machine learning algorithms: logistic regression, decision tree, naïve Bayes, random forest, SVM, and XGBoost.

We analyzed the relationship between ICB response and the tumor immune-microbiome landscape in STAD by examining the distribution of R and NR across INC and MC clusters. Significant differences in ICB response proportions were observed among subtypes, with notable variations in ICB scores across the three INC subtypes (Fig. 4a and b). A significant difference in ICB scores between R and NR groups (*P* < 0.05) emphasized the connection between immune infiltration and ICB response (Fig. 4c). A Sankey diagram visualized the correspondence among INC, MC, and ICB response classifications, suggesting interdependence among immune status, microbial composition, and therapeutic outcome (Fig. 4d). The UpSet plot visualizes the selected gene features for each fold, clearly demonstrating the overlap of gene features selected in each fold with those in other folds. This visualization highlights both the consistency and the differences in the gene selection process across folds (Fig. 4e). For microbial features, the top 10 genera based on relative abundance were selected. A correlation analysis was performed between the microbial genera and immune-related genes to explore potential microbe-gene regulatory associations relevant to ICB response. The heatmap illustrates several statistically significant correlations, with certain microbes (e.g., *Mycobacterium*, *Ralstonia*, *Desulfococcus*, and *Staphylococcus*) showing strong associations with key immune genes such as CD22, VIPR2, and FLT3. The corresponding correlation network provides a global view of the interaction landscape, where node size and color indicate the degree of connectivity, and edge thickness reflects the absolute Spearman’s correlation coefficient (Fig. 4f and g). This integrative visualization underscores the potential co-regulatory relationships between tumor-associated microbiota and host immune gene expression, suggesting a complex and possibly functional interplay within the tumor immune-microbial microenvironment. We developed integrated models by combining microbial and immune gene features using six machine learning algorithms. In most cases, the synergistic effects of microbial and immune gene features enhanced the differentiation of ICB response. However, the integrated models did not consistently outperform the single-modality models. We present the AUC performance of models using gene, microbe, and combined data to clearly illustrate the critical role of the synergistic effect between microbes and immune genes in predicting ICB response (Fig. 4h).

### Drug sensitivity profiling reveals immunophenotype-associated therapeutic vulnerabilities in STAD

**Fig 5:**
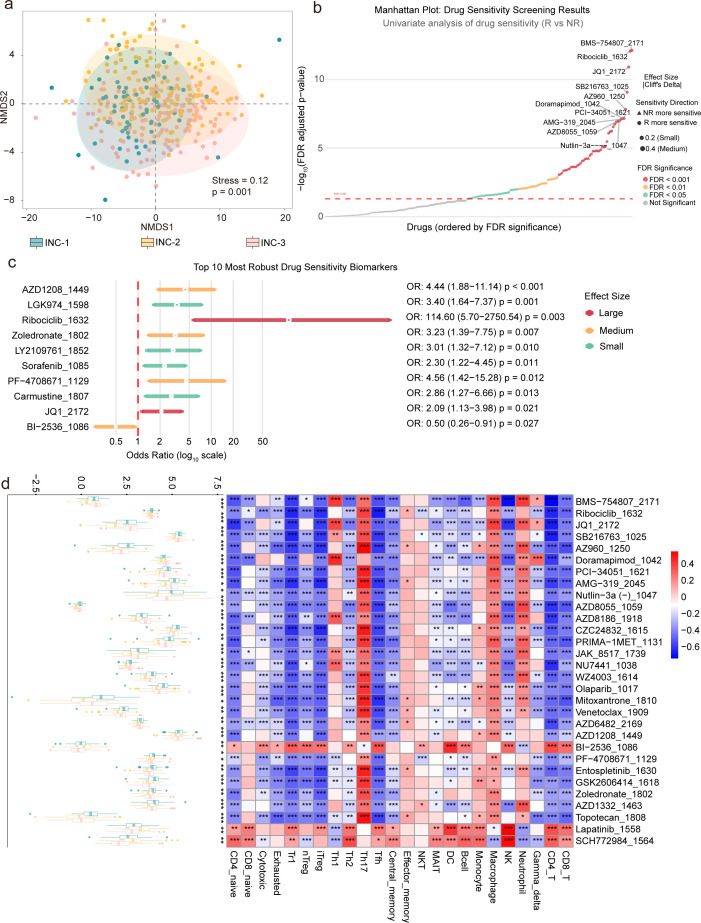
Analysis of drug sensitivity and potential therapeutic targets associated with immunophenotypes in STAD. (**a**) NMDS analysis illustrates the differential distribution patterns of drug sensitivity across immune-related NMF clusters (INC) subtypes. (**b**) Manhattan plot displaying statistical significance and effect sizes (Cliff’s delta) for 198 compounds, highlighting 106 compounds with significant differences in drug sensitivity between ICB R and NR after FDR correction. (**c**) Forest plot showing the top 10 most robust biomarkers associated with drug sensitivity and ICB response, identified via multivariate logistic regression adjusting for confounders such as INC subtype, tumor purity, and tumor stage. (**d**) Boxplots depicting the differences of the top 30 drugs among INC subtypes, alongside a correlation heatmap illustrating the associations between INC subtypes and 24 immune cell types.

Drug sensitivity profiling of 198 compounds from the GDSC database uncovered distinct pharmacologic vulnerabilities across INC subtypes. NMDS based on Bray-Curtis distances (stress = 0.18) revealed a clear separation of drug response profiles by INC subtypes (Fig. 5a). Univariate analysis identified 106 compounds with statistically significant differences in drug sensitivity between ICB R and NR after FDR correction, with effect sizes (Cliff’s delta) ranging from −0.327 to 0.578. Among these, 4 compounds exhibited large effects, 24 compounds showed medium effects, and 78 compounds showed small effects. The Manhattan plot displays both statistical significance and effect sizes for all 198 compounds, highlighting prominent candidates such as BMS-754807_2171, Ribociclib_1632, JQ1_2172, and SB216763_1025 (Fig. 5b). Multivariate logistic regression, adjusting for potential confounders including INC subtype, tumor purity, and tumor stage, identified 14 compounds that maintained significant associations with ICB response after covariate adjustment. The forest plot (Fig. 5c) illustrates the top 10 most robust biomarkers associated with drug sensitivity in response to immunotherapy. Among the most prominent biomarkers, AZD1208_1449 shows a strong association with treatment response, with an odds ratio (OR) of 4.44 (95% CI: 1.88–11.14), indicating that higher expression of AZD1208_1449 is 4.44 times more likely to predict a positive therapeutic outcome. Similarly, LGK974_1598 (OR: 3.40, 95% CI: 1.64–7.37) and Ribociclib_1632 (OR: 114.60, 95% CI: 5.70–2750.54) demonstrate a strong positive relationship with treatment success, with Ribociclib_1632 showing an extraordinarily high odds ratio, suggesting its potential as a powerful predictive biomarker for drug efficacy. These biomarkers, identified through multivariate analysis, exhibit consistent predictive value across both univariate and multivariate models, emphasizing their robustness as indicators of drug response in immunotherapy. Additionally, heatmaps of drug sensitivity ordered by INC and MC subtypes revealed clear, subtype-specific sensitivity patterns (Fig. S5 and S6). This pattern was further confirmed by significant correlations between these top compounds and the infiltration levels of 24 immune cell types (Fig. 5d). These results suggest that the tumor’s immune contexture plays an intrinsic role in shaping specific drug response patterns, providing insight into immune phenotype-associated therapeutic vulnerabilities.

## DISCUSSION

Over the past decade, the TIME has become a key factor in prognosis and therapeutic response in cancers like STAD (43). Studies show substantial heterogeneity in ICI patterns across patients with STAD, linked to molecular subtypes, prognosis, and ICB therapy response (44–46). Thorsson et al. (47) systematically classified the immune landscape across 33 cancer types, including STAD, identifying six immune subtypes with distinct cellular composition and survival outcomes. Similarly, Pang et al. revealed that immune-related gene signatures in gastric cancer could stratify patients with different survival probabilities and ICI characteristics (48). Recent research also emphasizes the role of the tumor microbiome in modulating immune responses and cancer progression (49). Microbial diversity and specific taxa are associated with ICI, inflammatory signaling, and ICB efficacy (50–52). Despite these insights, few efforts have comprehensively integrated immune, microbial, and transcriptomic data in STAD to develop unified subtyping frameworks or predictive models for ICB response. Moreover, while machine learning approaches have been applied to immune gene signatures or microbiome data individually, their combined predictive potential in STAD remains largely unexplored.

The TIME in STAD was comprehensively profiled by integrating immune infiltration patterns, immune gene signatures, microbial communities, response to ICB, and drug sensitivity landscapes. Using NMF, we identified three distinct immune subtypes (INC-1, INC-2, and INC-3), each with unique immune profiles and prognostic implications. These findings underscore the immunological heterogeneity of STAD and highlight the potential of immune-based stratification to inform personalized immunotherapeutic strategies (53–55). By analyzing DEIRGs across INC subtypes, we revealed that these genes are involved in diverse BPs, particularly cytokine signaling, cell–cell communication, and inflammatory responses. KEGG pathway enrichment further demonstrated strong associations with immune-regulatory signaling pathways such as TNF, MAPK, JAK-STAT, and TCR signaling, suggesting a dynamic immune regulatory network potentially influencing tumor progression and therapeutic responsiveness. Significant differences in immune checkpoint molecules (LAG3, PD-L1, CTLA4, TIGIT, and PD-1) across INC subtypes further support the link between immune infiltration patterns and ICB therapy sensitivity. These findings emphasize the potential for immune-based stratification to inform personalized immunotherapy (56, 57).

Our microbiome-based subtyping further refined the immunological stratification by defining three MCs (MC-1, MC-2, and MC-3). The differences in microbial diversity and composition across these subtypes—and their correlation with immune cell abundances—support the hypothesis that specific microbial populations may influence or reflect the immune microenvironment in STAD (58). The integration of immune and microbial clusters, as illustrated by chord diagrams and correlation networks, revealed potential crosstalk that warrants further mechanistic investigation (59). This integrative stratification strategy highlights a novel methodological innovation in tumor immune profiling: rather than analyzing immune or microbial features in isolation, we captured a more complete picture of TIME by jointly considering immune cell context, microbial ecology, and transcriptomic regulation. Such cross-domain integration has rarely been applied in STAD and represents a framework for decoding tumor–immune–microbe interactions.

The analysis of ICB response revealed significant associations between both immune and microbial subtypes with ICB responsiveness and scores. Using Boruta feature selection, we identified gene features with predictive value. Multi-omic models, combining the top 10 microbes and the top 20 immune genes, outperformed most single-omics classifiers across six machine learning algorithms (including logistic regression, random forest, decision tree, Naïve Bayes, SVM, and XGBoost). As shown in the AUC comparison (Fig. 4h), the integrated feature model achieved the highest performance, surpassing the models based on microbiome-only or gene-only features. For example, the AUC of logistic regression improved from 0.526 (microbiome-only) and 0.884 (gene-only) to 0.890 (combined model), while XGBoost’s AUC increased from 0.517 and 0.851 to 0.863. These results emphasize the complementary predictive value of integrating immune gene expression and microbial features in forecasting ICB responsiveness, providing strong support for the development of multi-omic biomarkers in clinical immuno-oncology. Additionally, drug sensitivity analysis revealed pharmacological heterogeneity among immune subtypes, with compounds such as BMS-754807, Ribociclib, SB216763, and JQ1 demonstrating subtype-specific efficacy and differential sensitivity between ICB R and NR (60, 61). These findings provide a rationale for leveraging immune subtypes to guide precision pharmacotherapy in STAD and underscore the need for immune-informed drug development strategies.

Despite the strengths of our integrative approach, several limitations exist. First, our study relies on bulk transcriptomic and microbial abundance data from public databases like TCGA, lacking the resolution of single-cell or spatial multi-omics. Second, microbiome profiles were inferred from sequencing data, which may introduce compositional biases. Third, while our models showed robust predictive performance, prospective validation in independent cohorts and clinical trials is needed. Exploring immune-microbial landscape dynamics during treatment could further inform decision-making and response monitoring.

## Data Availability

Data sets are available from TCGA. The following is the link to the data used in this research: https://portal.gdc.cancer.gov/projects/TCGA-STAD and https://github.com/knightlab-analyses/mycobiome.

## SUPPLEMENTAL MATERIAL

Supplemental material spectrum.02151-25-s0001.docx Figures S1 to S6.

Table S1 spectrum.02151-25-s0002.csv The ImmPort gene list.

Table S2 spectrum.02151-25-s0003.csv A complete table of microbe-immune association with q-values.

Table S3 spectrum.02151-25-s0004.csv The AUC results for each model, including the AUC, standard deviation, and 95% confidence interval for gene-only, microbe-only, and combined models.

Table S4 spectrum.02151-25-s0005.csv Summary of *P*-values, FDR-adjusted *P*-values, and Cliff's delta values of all drug univariate variables.

Table S5 spectrum.02151-25-s0006.csv Summary of the results of multivariate logistic regression.

Table S6 spectrum.02151-25-s0007.csv Per-sample table of predicted drug responses.

ASM does not own the copyrights to Supplemental Material that may be linked to, or accessed through, an article. The authors have granted ASM a non-exclusive, world-wide license to publish the Supplemental Material files. Please contact the corresponding author directly for reuse.
