# Supplementary material for: Integrative analysis of immune and microbial subtypes predicts immunotherapy response in stomach adenocarcinoma
Source: Microbiol Spectr. 2026 Feb 9;14(3):e02151-25. doi: 10.1128/spectrum.02151-25 (PMC12955421; doi:10.1128/spectrum.02151-25)
Supplement: Supplemental material — Figures S1 to S6. [file spectrum.02151-25-s0001.docx]

Integrative Analysis of Immune and Microbial Subtypes Predicts Immunotherapy Response in Stomach Adenocarcinoma

Yumeng Zhang^1^, Huakai Wen^1^, Xianfang Tang^2^, Yuhua Yao^1,3,4,*^

^1^School of Mathematics and Statistics, Hainan Normal University, Haikou 570100, China;

^2^School of Computer Science and Artificial Intelligence, Wuhan Textile University, Wuhan 430200, China;

^3^Key Laboratory of Data Science and Intelligence Education, Ministry of Education, Hainan Normal University, Haikou 570100, China;

^4^Key Laboratory of Computational Science and Application of Hainan Province, Hainan Normal University, Haikou 570100, China

*Correspondence: Yuhua Yao, Email: yaoyuhua@hainnu.edu.cn

**TCGA decontamination**

Although TCGA protocols did not include contamination controls during the processing of their samples, we showed that in silico methods could be used to decontaminate the TCGA bacteriome. The fundamental principle of these methods is that consistent negative correlations exist for external (e.g., reagent, environmental) contaminating taxa between their read fractions and analyte (DNA or RNA) concentrations (*1*). A published tool named decontam (https://github.com/benjjneb/ decontam) (version 1.14.0) (*1*) wraps the method into anRpackageand function based on two underlyingmathematical assumptions: (i) the contaminants are added in uniform amounts across samples; and (ii) the amount of contaminant DNA or RNA is small relative to the true sample DNAor RNA (microbial or host). Since per-sample DNA and RNA concentrations are available in TCGA metadata, they can be used to indicate putative contaminating taxa. Importantly, though, our past analyses demonstrated that too stringent of an in silico decontamination threshold actually removes flora known to be associated with a given body site (e.g., too stringent decontamination of NAT colon tissues in TCGA dissociates it from normally-associated fecal material). Additionally, there are difficulties of strict filtering with taxa that are known commensals and/or pathogens but also can be contaminants in certain contexts, even at the species level (e.g., Malassezia restricta, a skin fungus). Thus, in our mycobiome analyses, we sought a balance between strict filtering, allowance of known commensals/pathogens, inclusion of WIS-identified (this study) or HMP-identified (*2*) fungi, and inclusion of fungi of unknown significance that may be related to cancer biology.

Decontamination was thus broken into two steps: (i) Statistical decontamination via decontam using per-sample DNA or RNA concentrations and read fractions across plate-center batches (see below); and (ii) manual curation, comparison against WIS-identified and HMP-identified fungi, and literature review prior to making final determinations.

Step #1: TCGAsample identifiers (e.g., ‘‘TCGA-02-0001-01C-01D-0182-01’’) denoted the sequencing center and plate within that center upon which the sample was run (for details, see https://docs.gdc.cancer.gov/Encyclopedia/pages/ TCGA_Barcode/). These barcodes were used to extract all sequencing plate-center combinations using the last two sets of integers (e.g.,‘‘0182-01’’ is plate 182 from center 1). We previously found the plate-center method to work well on TCGA bacterial data, as it removed many likely contaminants while retaining several known commensals and pathogens. Since decontam effectively performs a regression analysis to determine if a taxon is a contaminant, we requiredR10 samples per plate-center batch, retaining 329 total plate-center batches among samples positive for fungi. Decontam was then run in ‘‘frequency’’ mode, identifying putative contaminants using TCGA sample aliquot concentrations, a default P* stringency threshold of 0.1, and the default batch.combine=‘‘minimum’’ parameter, such that a taxon was removed if identified in any one of the 329 plate-center batches as a contaminant. This analysis identified 57 putative contaminants out of 319 total fungi with R1 reads identified during direct genome alignments. Table S3 summarizes the decontam output and contaminant predictions.

Step #2: All 319 fungal taxa found in TCGA were cross-referenced against species identified in the WIS tumor mycobiome cohort (this study), the HMP gut mycobiome cohort (*2*), and 131 other papers in the literature. This comprehensive literature survey informed the final decontamination decisions. Specifically, the following decision making process was applied: (i) Any fungal specie identified in the WIS tumor mycobiome cohort or HMP gut mycobiome cohort was retained; (ii) any fungal species known in the literature to have caused a clinically pathogenic infection or be a human commensal was retained; (iii) any fungal species with evidence of no known human association was discarded; (iv) any species that had little evidence for or against human associations (i.e., ‘‘unknown’’ human associations) had their fate decided by the plate-center decontam predictions. This process ultimately discarded 95 species (29.8% of total) as contaminants, comprising 2.1% of total reads, and retained 224 species as non-contaminants.


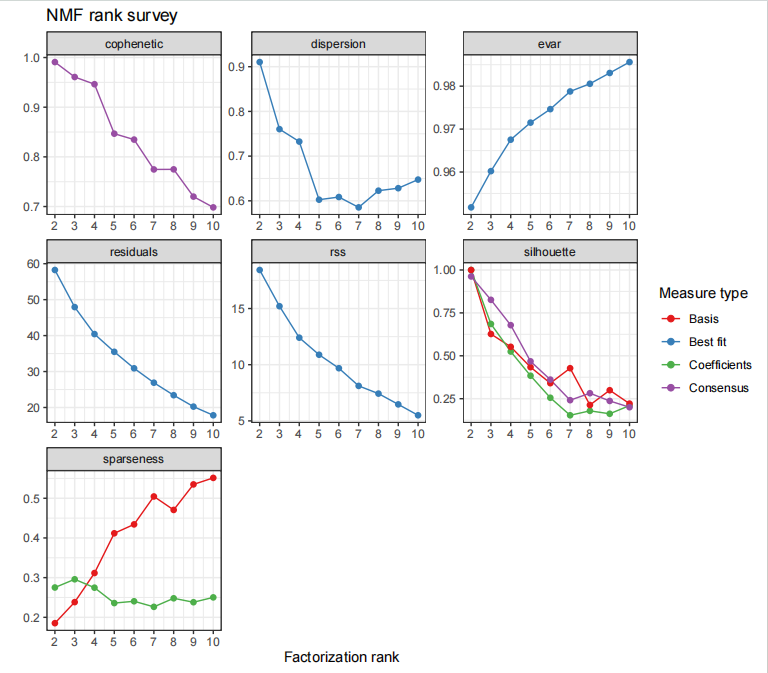


**Supplementary Figure 1.** Model selection for NMF clustering. Multiple diagnostic metrics are plotted against a range of factorization ranks (k from 2 to 10). The optimal rank was determined to be k=3, as it represents the point with a high cophenetic coefficient (indicating model stability) prior to a marked decrease, and the elbow of the residuals curve (indicating a balance between model fit and complexity).


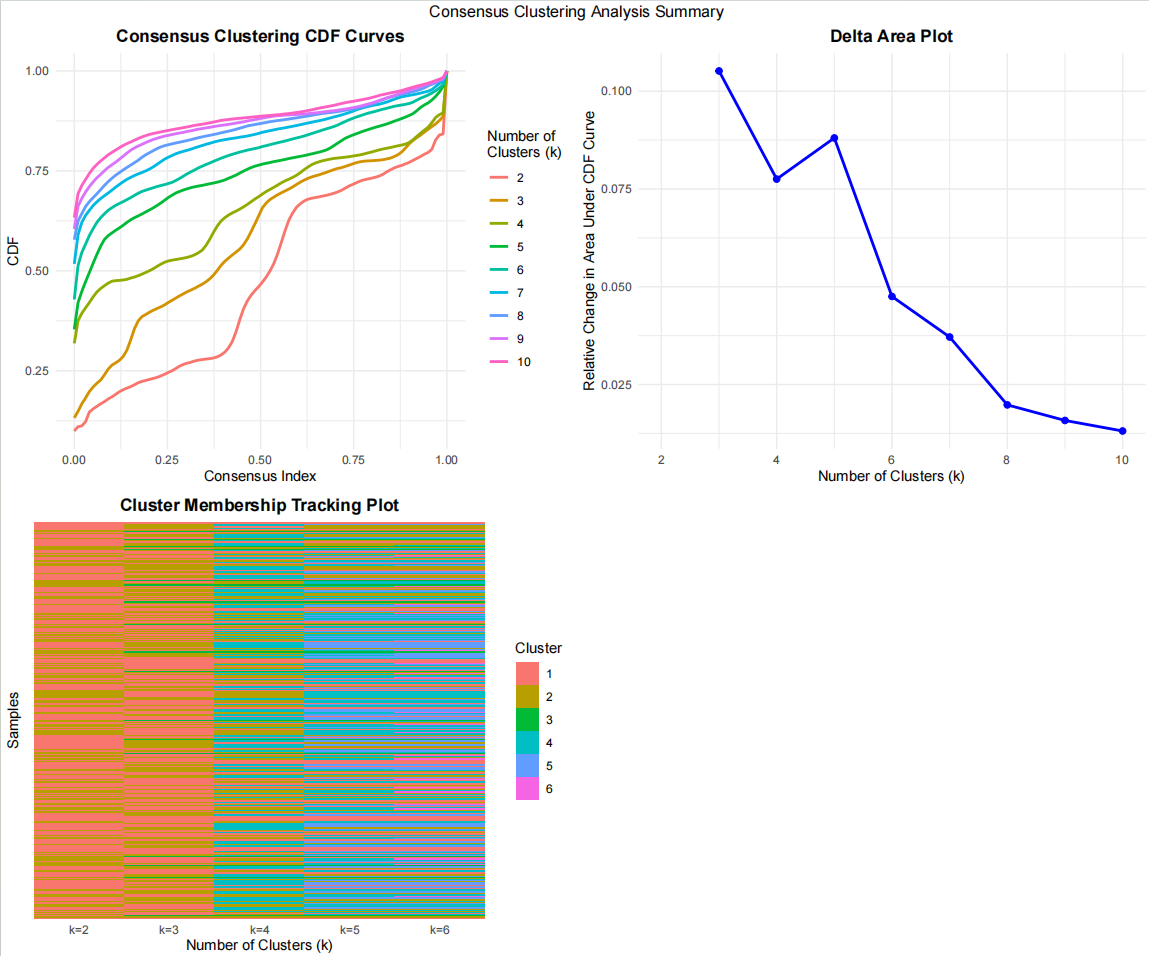


**Supplementary Figure 2.** Consensus Clustering Analysis. Consensus Clustering CDF Curves: The cumulative distribution function (CDF) curves for different numbers of clusters (k = 2 to 10) are shown, indicating the consensus index for each clustering solution. Delta Area Plot: The relative change in the area under the CDF curve is plotted against the number of clusters, highlighting the most stable clustering solution (k = 3), where the greatest change in the delta area is observed. Cluster Membership Tracking Plot: The assignment of samples to clusters is visualized across different k values, with distinct cluster membership patterns for each number of clusters (k = 2 to 6).


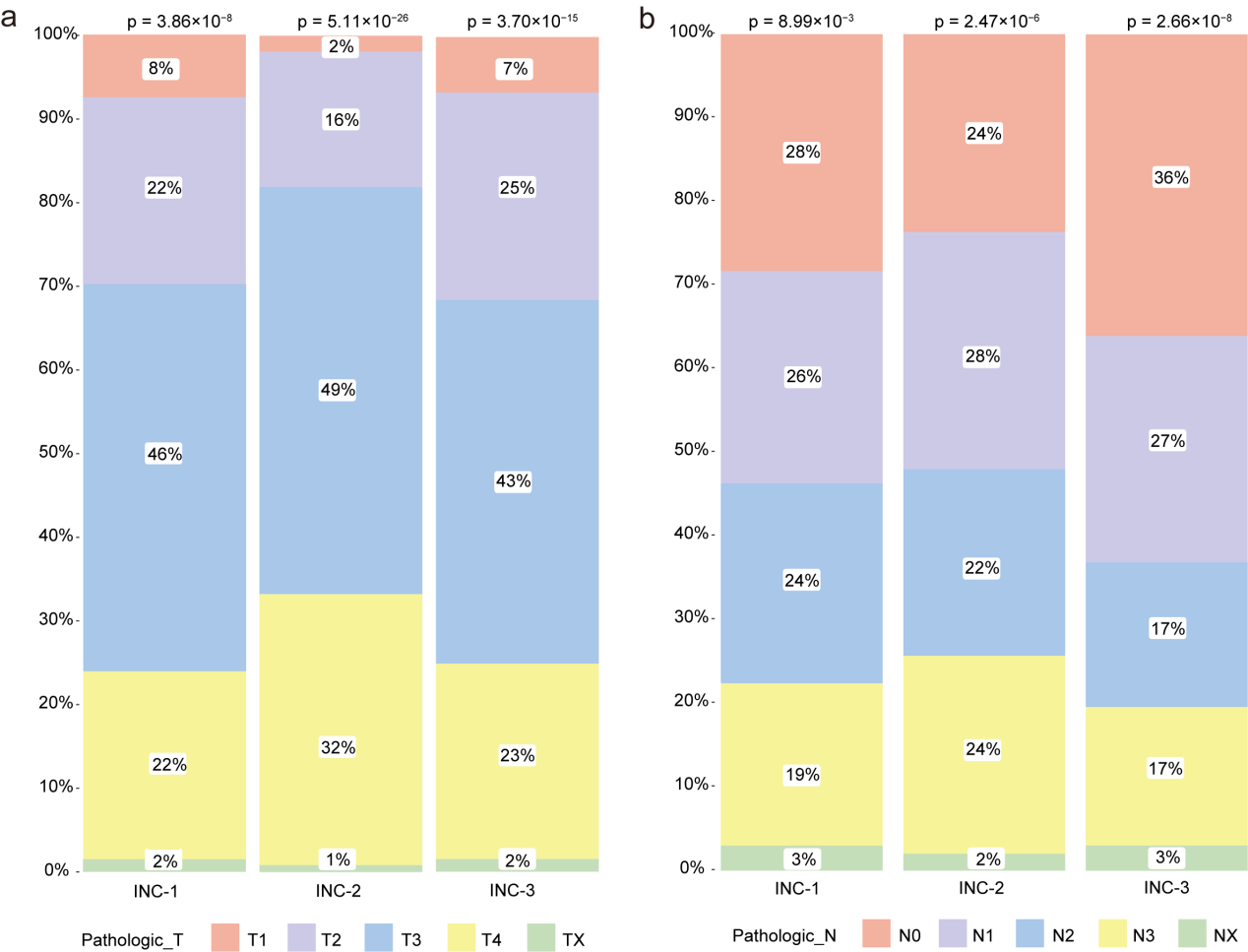


**Supplementary Figure 3.** Distribution of pathological T and N stages among distinct immune cell infiltration (INC) groups. Bar plots illustrate the relative proportions of T stages (T1–T4, TX) and N stages (N0–N3, NX) within each INC group. Statistical differences between groups were assessed, with corresponding p-values indicated.

**
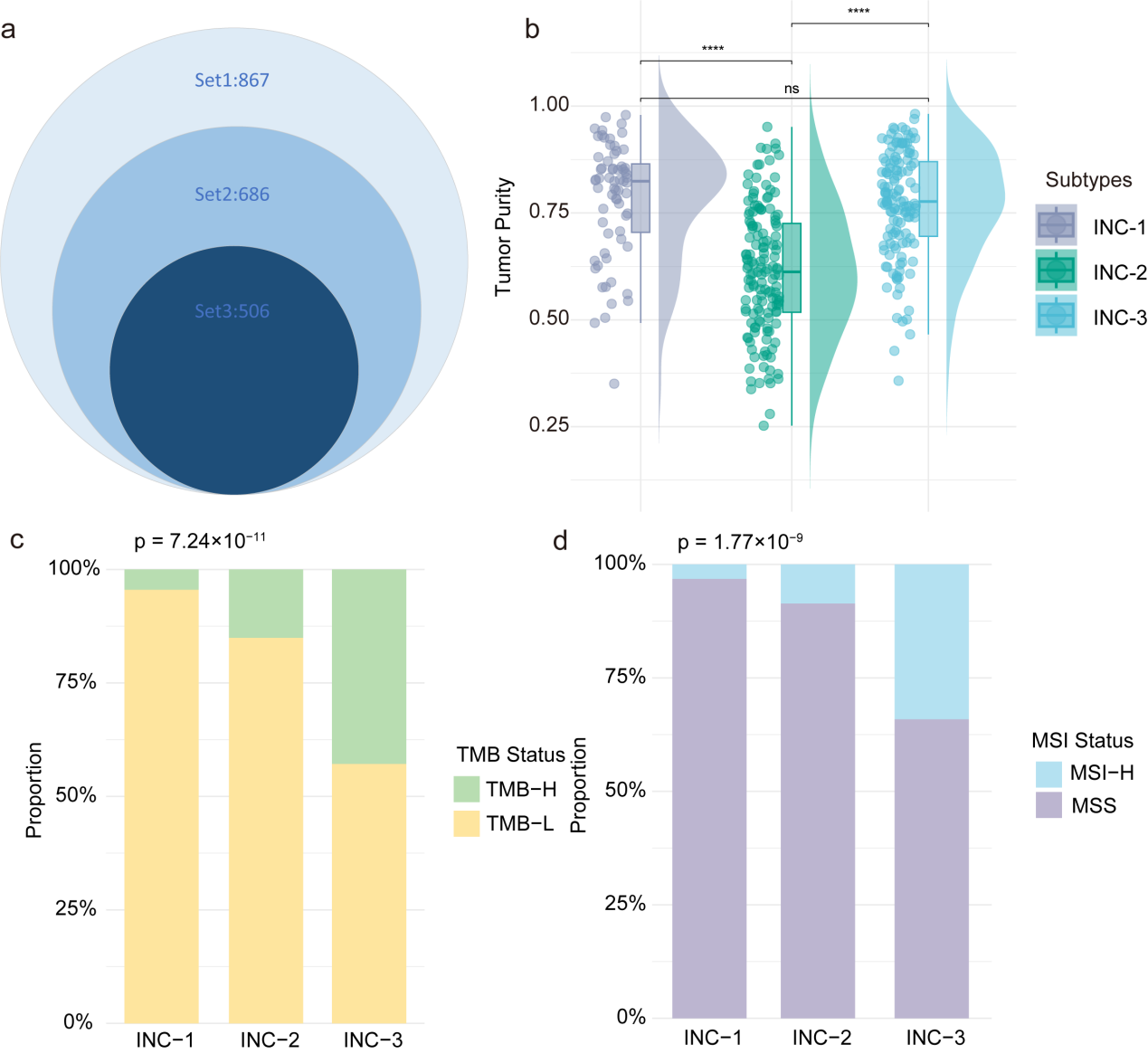
**

**Supplementary Figure 4. Differential analysis of gene expression, tumor purity, TMB, and MSI across INC-1, INC-2, and INC-3 subtypes.** (a) Venn diagram depicting the gene sets identified by differential expression analysis across INC-1, INC-2, and INC-3 subtypes. (b) Box plot comparing tumor purity expression across INC-1, INC-2, and INC-3 subtypes. (c) Bar plot illustrating the differences in TMB status between INC-1, INC-2, and INC-3 subtypes. (d) Bar plot comparing the MSI status across INC-1, INC-2, and INC-3 subtypes.


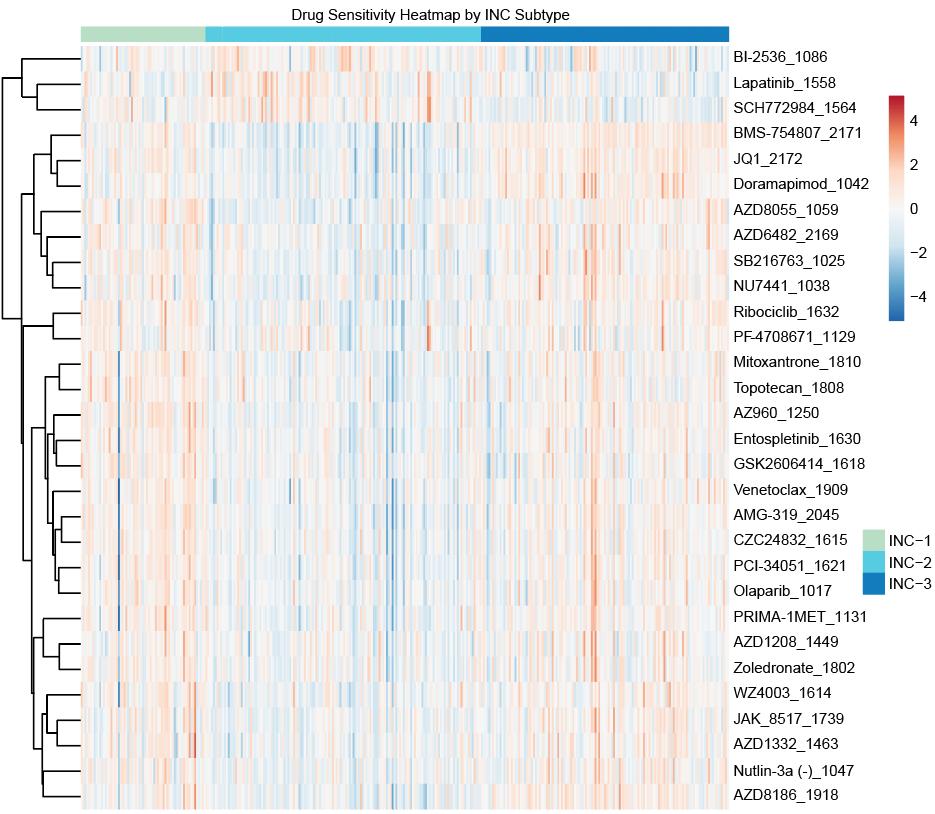


**Supplementary Figure 5. Heatmap of molecular expression for the top 30 drugs across INC subtypes.**


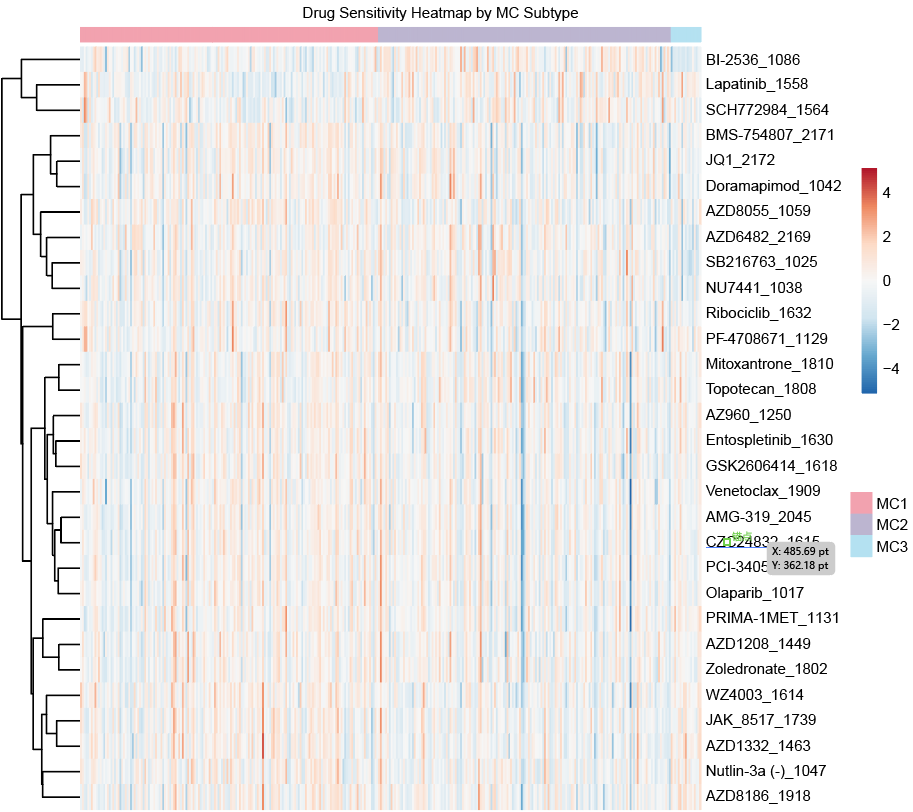


**Supplementary Figure 6. Heatmap of molecular expression for the top 30 drugs across MC subtypes.**

1. N. M. Davis, D. M. Proctor, S. P. Holmes, D. A. Relman, B. J. Callahan, Simple statistical identification and removal of contaminant sequences in marker-gene and metagenomics data. *Microbiome* **6**, 226 (2018).

2. A. K. Nash *et al.*, The gut mycobiome of the Human Microbiome Project healthy cohort. *Microbiome* **5**, 153 (2017).
